# Epigenetic Engineering of Ribosomal RNA Genes Enhances Protein Production

**DOI:** 10.1371/journal.pone.0006653

**Published:** 2009-08-14

**Authors:** Raffaella Santoro, Philipp Lienemann, Martin Fussenegger

**Affiliations:** Department of Biosystems Science and Engineering, ETH Zurich, Basel, Switzerland; Deutsches Krebsforschungszentrum, Germany

## Abstract

Selection of mammalian high-producer cell lines remains a major challenge for the biopharmaceutical manufacturing industry. Ribosomal RNA (rRNA) genes encode the major component of the ribosome but many rRNA gene copies are not transcribed [1]–[5] due to epigenetic silencing by the nucleolar remodelling complex (NoRC) [6], which may limit the cell's full production capacity. Here we show that the knockdown of TIP5, a subunit of NoRC, decreases the number of silent rRNA genes, upregulates rRNA transcription, enhances ribosome synthesis and increases production of recombinant proteins. However, general enhancement of rRNA transcription rate did not stimulate protein synthesis. Our data demonstrates that the number of transcriptionally competent rRNA genes limits efficient ribosome synthesis. Epigenetic engineering of ribosomal RNA genes offers new possibilities for improving biopharmaceutical manufacturing and provides novel insights into the complex regulatory network which governs the translation machinery in normal cellular processes as well as in pathological conditions like cancer.

## Introduction

On the way from DNA to product translation is a major bottleneck which may limit the specific productivity of mammalian production cell lines. Cells can upregulate the rate of protein synthesis either by increasing the translational efficiency of existing ribosomes or by increasing the capacity of translation through the production of new ribosomes (ribosome biogenesis) [7]. With about 80% of total nuclear transcription being dedicated to the synthesis of ribosomal RNA (rRNA), ribosome biogenesis is one of the major metabolic activity of mammalian cells. Ribosome assembly occurs within the nucleolus and requires coordinated expression of four rRNAs (45S pre-rRNA, which is subsequently processed into 18S, 5.8S, 28S and 5S rRNA) and about 80 ribosomal proteins (r-proteins) [8]. 45S pre-rRNA is transcribed in the nucleolus by polymerase I (Pol I), 5S RNA is transcribed by Pol III at the nucleolar periphery and then imported into the nucleolus [9] and r-proteins are transcribed by Pol II. Thus, ribosome biogenesis requires orchestration of transcription by different polymerases operating in different compartments. In mammalian cells, these processes are largely unknown.

Transcription of 45S pre-rRNA is the key step of ribosome biogenesis. Mammalian haploid genomes contain about 200 ribosomal RNA genes of which only a fraction is transcribed at any given time, while the rest remains silent [4], [10]. Active and silent genes are distinct with respect to chromatin configuration: active genes have a euchromatic structure, whereas silent genes are heterochromatic. The promoter of active rRNA genes is free of CpG methylation and is associated with acetylated histones. The opposite is true of silent genes [1], [2], [11]. Recent results indicate that the key determinant of rDNA silencing is NoRC (nucleolar remodeling complex). NoRC consists of TIP5 (TTF-1-interacting protein 5) and the ATPase SNF2 h [6]. NoRC binds to the rDNA promoter of silent genes and represses rDNA transcription through histone-modifying and DNA-methylating activities [1]–[4].

## Results

**Figure 1:**
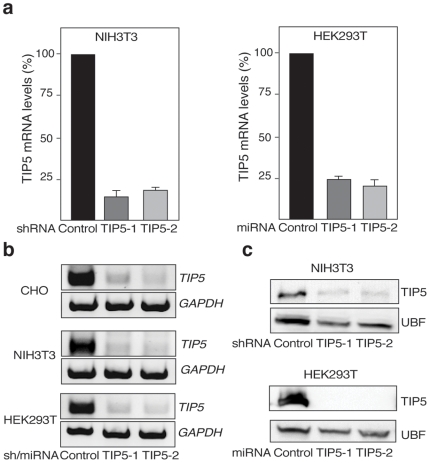
TIP5 knockdown. a. qRT-PCR of TIP5 mRNA of NIH/3T3 cells stably expressing shRNA-TIP5-1 and TIP5-2 sequences and of HEK293T cells stably expressing miRNA-TIP5-1 and TIP5-2 sequences. Data were normalized to GAPDH mRNA levels. Data are from two independent experiments. b. Semiquantitative RT-PCR of TIP5 mRNA of stable shRNA-TIP5-1/2 NIH/3T3, miRNA-TIP5-1/2 HEK293T and miRNA-TIP5-1/2 CHO-K1 cells. As control, RT-PCR of GAPDH mRNA is shown. Data are from two independent experiments. c. TIP5-specific Western blot analysis of stable shRNA-TIP5-1/2 NIH/3T3 and miRNA-TIP5-1/2 HEK293T cells. CHO-K1 cells could not be analyzed because the anti-TIP5 antibody does not recognize hamster TIP5. To normalize protein loading, the levels of UBF were monitored using an anti-UBF antibody.

The presence of transcriptionally silent rRNA genes raises the question as to whether this represents a limiting factor for the synthesis of rRNA and the production of ribosomes. It has been hypothesized that cells can modulate rDNA transcription levels by altering the transcriptional activity of each gene and/or by altering the number of active genes [12]. However, a satisfying correlation between 45S pre-rRNA synthesis levels and the number of rRNA genes has not been found. For instance, in *S. cerevisiae*, reducing the number of rRNA genes by about two thirds did not affect total rRNA production [13]. Similarly, maize inbred lines and aneuploid chicken cells, containing different numbers of rRNA copies displayed the same levels of rRNA transcription [14], [15]. With the aim of engineering cells for increased synthesis of recombinant proteins, we determined whether a decrease in the number of silent rRNA genes enhances 45S pre-rRNA synthesis and, as consequence, also stimulates ribosome biogenesis and increases the number of translation-competent ribosomes. Therefore, we used RNA interference to knock down TIP5 expression and constructed stably transgenic shRNA-expressing NIH/3T3 or miRNA-expressing HEK293T and CHO-K1 using shRNA/miRNA sequences specific for two different regions of TIP5 (TIP5-1 and TIP5-2). Stable cell lines expressing scrambled shRNA and miRNA sequences were used as control. There were two reasons for producing stable cell lines rather than performing transient transfections with plasmids expressing shRNA-TIP5 or miRNA-TIP5 sequences. First, the loss of repressive epigenetic marks like CpG methylation is a passive mechanism, requiring multiple cell divisions. Second, even though HEK293T cells can be transfected relatively easily, the poor transfection efficiency of NIH/3T3 and CHO-K1 cells would compromise subsequent analyses of endogenous rRNA, ribosome levels and cell growth properties. To determine the efficiency of TIP5 knockdown in the selected clones, we measured TIP5 mRNA levels by quantitative and semiquantitative reverse-transcriptase-mediated PCR (**Fig. 1a,b**). TIP5 expression decreased about 70–80% in NIH/3T3/shRNA-TIP5-1 and -2 cells when compared to control cells (**Fig. 1a**). A similar reduction in TIP5 mRNA levels was observed in stable HEK293T. TIP5 mRNA levels in CHO-K1-derived cells could be measured only by semiquantitative PCR (**Fig. 1b**) but the reduction of TIP5 mRNA was similar to that of stable NIH/3T3 and HEK293T cells. Consistent with this, Western blot analysis of stable NIH/3T3 and HEK293T cells indicated a strong reduction of TIP5 protein levels (**Fig. 1c**). These results demonstrate that the established cell lines contain low levels of TIP5.

**Figure 2:**
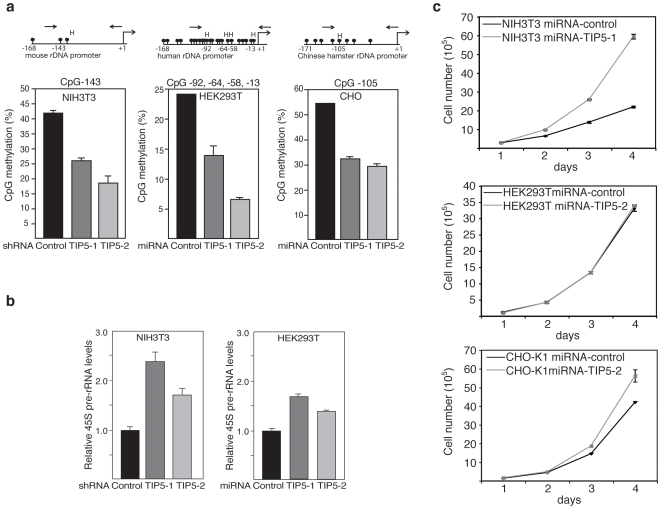
TIP5 depletion enhances rRNA production. a. Depletion of TIP5 decreases CpG methylation of rDNA promoters. Diagram of a mouse, human and Chinese hamster rDNA promoter regions including the HpaII (H) sites analyzed. Black circles indicate CpG dinucleotides. Arrows represent the primers used to amplify HpaII-digested DNA. rDNA CpG methylation levels were measured in NIH/3T3, HEK293T and CHO-K1 cells stably expressing shRNA- and/or miRNA-TIP5-1/2 and control sequences. Data represent the amounts of HpaII-resistant rDNA normalized to the total rDNA calculated by amplification with primers encompassing DNA sequences lacking HpaII-sites and undigested DNA. Data are from two independent experiments. b. Depletion of TIP5 enhances rRNA synthesis. qRT-PCR-based 45S pre-rRNA levels of stable NIH/3T3 and HEK293T cell lines were normalized to GAPDH mRNA levels. Data are from two independent experiments. c. Growth curves of NIH/3T3, HEK293T and CHO-K1 cells stably expressing miRNA-TIP5 and control sequences. Data are from two independent experiments.

CpG methylation of the mouse rDNA promoter impairs binding of the basal transcription factor UBF, and the formation of preinitiation complexes is prevented [11]. In NIH/3T3 cells about 40% to 50% of rRNA genes contain CpG-methylated sequences and are transcriptionally silent [11]. The sequences and CpG density of the rDNA promoter in humans, mice and Chinese hamsters differ significantly. In humans, the rDNA promoter contains 23 CpGs, while in mice and Chinese hamsters there are 3 and 8 CpGs, respectively (**Fig. 2a**). To verify that TIP5 knockdown affects rDNA silencing, we determined the rDNA methylation levels by measuring the amount of meCpGs in the CCGG sequences. Genomic DNA was HpaII-digested, and resistance to digestion (i.e. CpG methylation) was measured by quantitative real-time PCR using primers encompassing HpaII sequences (CCGG) [1]. There was a decrease in CpG methylation within the promoter region of a the majority of rRNA genes in all TIP5 knock-down cell lines, underscoring the key role of TIP5 in promoting rDNA silencing (**Fig. 2a**). To determine whether a decrease in the number of silent genes affects the amounts of the rRNA transcript, we measured 45S pre-rRNA synthesis by qRT-PCR using primers that encompassed the first rRNA processing site. As expected, in both TIP5-depleted NIH/3T3 and HEK293T cells, an enhancement of rRNA production was detected (**Fig. 2b**). rRNA transcription in CHO-K1 cells could not be determined as the rRNA coding sequence is not available for this cell line. These results indicate that TIP5 knockdown and a decrease in rDNA silencing enhances rRNA production.

Synthesis of rRNA is the first event in ribosome biogenesis and is tightly regulated by the activity of cellular metabolism [8], [16]. Conditions compromising cellular metabolism such as starvation, toxic lesions, aging, viral infections and cancer downregulate rDNA transcription. Conversely, rDNA transcription is upregulated upon reversal of such conditions and by agents that stimulate growth. To determine whether a decrease in the number of silent rRNA genes affects cellular proliferation, we compared the proliferation rate of TIP5-depleted cells and control cells (**Fig. 2c**). Previous data indicated that cells overexpressing TIP5 grow more slowly than parental cell lines [17]. Here we show that both NIH/3T3 and CHO-K1 cells, expressing miRNA-TIP5 sequences, proliferate at a faster rate than the control cells, suggesting that a decrease in the number of silent rRNA genes does have an impact on cell metabolism. In contrast, TIP5 depletion in HEK293T did not significantly affect cell proliferation, probably because these cells had already reached their maximum rate of proliferation. These data indicate that depletion of TIP5 and a consequent decrease in rDNA silencing have the potential to enhance cell proliferation.

**Figure 3:**
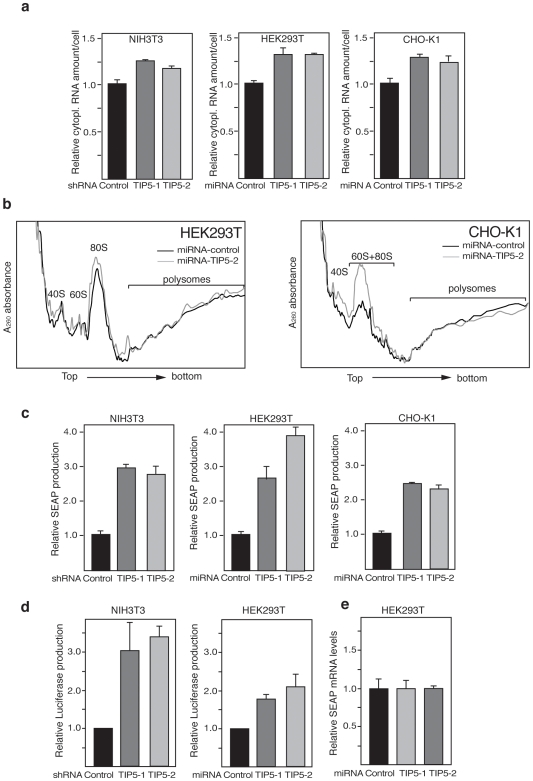
Depletion of TIP5 enhances ribosome synthesis. a. Relative amounts of cytoplasmic RNA/cell in stable NIH/3T3, HEK293T and CHO-K1 cells. Data represent the average of two experiments performed in triplicate. b. Polysome profile of stable HEK293T and CHO-K1 cell lines. The data show one representative experiment out of two independent experiments. c. Depletion of TIP5 enhances production of recombinant proteins. SEAP expression of stable NIH/3T3, HEK293T and CHO-K1 cell lines engineered with the constitutive SEAP expression vector pCAG-SEAP. Data are from three independent experiments. d. Luciferase expression of stable NIH/3T3 and HEK293T cell lines engineered with the constitutive luciferase expression vector pCMV-luciferase. Data are from two independent experiments. e. Relative SEAP transcript levels of stable HEK293T cells transfected with a constitutive SEAP expression vector. Values were normalized to GAPDH mRNA levels. Data are from two independent experiments.

In mammalian cell cultures, the rate of protein synthesis is an important parameter, which is directly related to the product yield. To determine whether depletion of TIP5 and a consequent decrease in rDNA silencing increases the number of translation-competent ribosomes in the cell, we initially measured the levels of cytoplasmic rRNA. In the cytoplasm, most of the RNA consists of processed rRNAs assembled into ribosomes. As shown in **Fig. 3a**, all TIP5-depleted cell lines contained more cytoplasmic RNA per cell, suggesting that these cells produce more ribosomes. Also, analysis of the polysome profile showed that TIP5-depleted cells contained more ribosome subunits (40S, 60S and 80S) compared to control cells (17.0±0.5% and 50.0±2%, in HEK293T and CHO-K1, respectively; **Fig. 3b**). To determine whether depletion of TIP5 and decrease in rDNA silencing enhance heterologous protein production, we transfected stable TIP5-depleted NIH/3T3, HEK293T and CHO-K1 derivatives with expression vector promoting constitutive expression of the human placental secreted alkaline phosphatase SEAP (pCAG-SEAP) or luciferase (pCMV-luciferase) (**Fig. 3c,d**). Quantification of protein production after 48 h revealed a two- to four-fold increase in both SEAP and luciferase production in TIP5-depleted cells compared to the control cell lines, indicating that TIP5-depletion increases heterologous protein production. As expected, no changes in reporter gene mRNA levels could be detected in stable HEK293T (**Fig. 3e**), NIH/3T3 and CHO-K1 cells (data not shown). All these results show that a decrease in the number of silent rRNA genes enhances ribosome synthesis and increases the potential of the cells to produce recombinant proteins.

**Figure 4:**
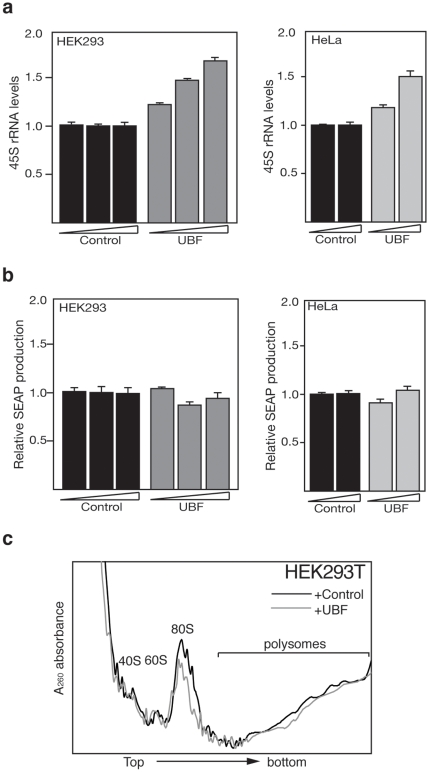
Enhancement of the rRNA transcription rate does not increase recombinant protein synthesis. a. qRT-PCR of 45S rRNA levels in HEK293T and HeLa following expression of increasing amounts of UBF (pCMV-UBF). rRNA levels were normalized to GAPDH mRNA quantities. Data are from two independent experiments. b. SEAP expression of HEK293T and HeLa cells co-transfected with pCAG-SEAP and either pCMV-UBF or pCMV-TAP-tag (control). Data are from two independent experiments. c. Polysome profile of HEK293T cells transfected with either pCMV-UBF or pCMV-TAP-tag (control). The data show one representative experiment out of two independent experiments.

Ribosome production requires coordinated expression and assembly of rRNAs and r-proteins. To determine whether an increase in the rate of 45S pre-rRNA transcription of each rRNA gene is sufficient to enhance ribosome production and heterologous protein synthesis, we transfected SEAP-expressing HEK293T and HeLa cells with a constitutive expression vector encoding the basal transcription factor UBF or the parental control vector. UBF binds to active rRNA genes, promotes transcription initiation and regulates the elongation rate [11], [18]. As expected, UBF stimulates 45S pre-rRNA synthesis in a dose-dependent manner (**Fig. 4a**). To determine whether a higher rRNA transcription rate enhances the synthesis of recombinant proteins, we profiled SEAP production. As shown in **Fig. 4b**, overexpression of UBF did not stimulate SEAP production in HEK293T and HeLa cells, suggesting that a higher synthesis rate of all rRNA genes is not enough to enhance heterologous protein production capacity of mammalian cells. Also, polysome profile analysis of UBF-overexpressing HEK293T cells, indicated a decrease in the ribosome content compared to the control cells (22.0±1.0%; **Fig. 4c**). This result suggests that enhancement of the rate of 45S pre-rRNA synthesis, mediated by overexpression of UBF, is not coordinated with ribosome biogenesis, thus probably generating dysfunctional and incomplete ribosomes. Our data demonstrate that a decrease in the number of transcriptionally silent rRNA genes stimulates ribosome production, while an increase in the rate of rRNA transcription did not.

## Discussion

Control of the translational machinery and an increase in the rate of cellular protein synthesis are major challenges in biopharmaceutical manufacturing. Different metabolic engineering strategies have been designed to improve the productivity of mammalian host cell lines, such as transcription engineering using trigger-inducible expression systems, translation engineering consisting of improving ribosomal entry and translation initiation and controlled proliferation technology which blocks proliferation at high cell density to allow an extended period of high productivity [19]–[22]. Here we show that engineering the epigenetic state of rRNA genes enhances the productivity of recombinant proteins. Depletion of TIP5 is a straightforward metabolic engineering strategy to increase production of protein pharmaceuticals, which may speed-up biopharmaceutical manufacturing and/or drive production economics into a viable range. To the best of our knowledge, this is the first time that an engineered decrease in the number of silent rRNA genes could be correlated with enhanced production of rRNA and ribosomes and consequently with higher productivity of mammalian cells. Thus, our data indicate a link between the amounts of transcriptionally competent rRNA genes with the metabolic activity of the cell. A sustained increase in the rates of protein synthesis is required during normal cellular process such as organ regeneration, but also under pathological conditions such as tumor development [23], [24]. Cancer cells upregulate rRNA transcription to meet the demand for the increased production of ribosomes and for protein synthesis of the rapidly proliferating tumors. It was recently shown that several tumor types contain rRNA genes with a low density of CpG methylation [25]–[27]. However, a correlation between rDNA hypomethylation and ribosome biogenesis has not been found. We therefore provide an explanation of how impairment of rDNA silencing contributes to the aberrant ribosome biogenesis that is essential for the active growth of the tumor. Our data link rRNA gene silencing, proliferation and productivity and so offer a new strategy to improve biopharmaceutical production of important protein therapeutics. They also provide new insights into the complex regulatory network that governs the translation machinery in normal cells and in pathological processes like cancer.

## Methods

### Plasmids

pCMV-UBF was kindly provided by Ingrid Grummt. pCMV-TAP-tag contains TAP-tag sequences transcribed under control of cytomegalovirus immediate early promoter.

### Stable cell lines

NIH/3T3 cells were stably transfected with plasmids expressing shRNA TIP5-1 (5′-GGACGATAAAGCAAAGATGTTCAAGAGACATCTTTGCTTTATCGTCC-3′) and TIP5-2 (5′-GCAGCCCAGGGAAACTAGATTCAAGAGATCTAGTTTCCCTGGGCTGC-3′) sequences under control of the H1 promoter. HEK293T and CHO-K1 cells were stably transfected with plasmids expressing control miRNA or miRNA sequences targeting TIP5 (TIP5-1: 5′- GATCAGCCGCAAACTCCTCTGAGTTTTGGCCACTGACTGACTCAGAGGATTGCGGCTGAT-3′; TIP5-2: 5′- GCAAAGATGGGATCAGTTAAGGGTTTTGGCCACTGACTGACCCTTAACTTCCCATCTTTG-3′) according to the Block-iT Pol II miR RNAi system (Invitrogen).

### Transcription analysis

45S pre-rRNA transcription was measured by qRT-PCR in accordance with the standard procedure and using the Universal Master mix (Diagenode). Primer sequences used to detect mouse and human 45S pre-rRNA and GAPDH have been described before [**1**], [**2**].

### CpG methylation analysis

Methylation of mouse and human rDNA was measured as described previously [**1**], [**28**]. Primers used for analysis of rDNA methylation in CHO-K1 cells were: -168/-149 forward 5′-GACCAGTTGTTGCTTTGATG-3′; -10/+10 reverse 5′-GCGTGTCAGTACCTATCTGC-3′; -100/-84 forward 5′-TCCCGACTTCCAGAATTTC-3′.

### Protein production

Protein production was assessed 48h after transfection of a constitutive SEAP (pCAG-SEAP) or luciferase expression vector (pCMV-Luciferase). SEAP production was measured by a p-nitrophenyphospate-based light-absorbance time course [29]. Luciferase profiling was performed according to the manufacturer's instructions (Applied biosystems, Tropix^®^ luciferase assay kit). Values were normalized to cell numbers and to transfection efficiency. Transfection efficiency was measured by flowcytometric analysis of cells transfected with a GFP expression vector (GFP-C1, Clontech). All experiments were performed in triplicate and were repeated three times.

### Growth curves

10^5^ cells were seeded per well of a 6-well plate and each day cells were trypsinized, collected and counted with Casy^®^ Cell Counter (Schaerfe System). All cell lines showed similar viability. Experiments were performed in duplicates and repeated twice.

### Polysome profile

Cells were treated with cycloheximide (100 µg/ml, 10 min) and lysed in 20 mM Tris-HCl, pH7.5, 5 mM MgCl_2_, 100 mM KCl, 2.5 mM DTT, 100 µg/ml cycloheximide, 0.5% NP40, 0.1 mg/ml heparin and 200 U/ml RNAse inhibitor at 4°C. After centrifugation at 8,000 g for 5 min, the supernatants were loaded onto a 15%–45% sucrose gradient and centrifuged for 4 h at 28,000 rpm at 4°C. 200 µl fractions were collected and the optical density of individual fractions was measured at 260 nm. Quantifications were performed by comparing the total absorbance corresponding to 80S (HEK293T) and 60-80S (CHO-K1) peaks.

### Cytoplasmatic RNA measurements

Cells were tripsinized and counted in triplicates with Casy^®^ Cell Counter (Schaerfe System). Triplicates of 10^6^ cells were washed with PBS, lysed in cytoplasmic buffer (10 mM HEPES, pH 7.6, 60 mM KCl, 1 mM EDTA, 0.075% Triton X-100, 1 mM DTT) and centrifuged at 8000 g. RNA was isolated from the lysate supernatants using TRIzol^®^ (Invitrogen) according to the manufacturer's instructions and quantified in triplicate by measuring absorbance at 260 nm.

### Antibodies

The Anti-TIP5 antibody was purchased from Diagenode, the Anti-UBF antibody from Santa Cruz.
